# Preparedness for *Candida auris* in Canadian Nosocomial Infection Surveillance Program (CNISP) hospitals, 2024

**DOI:** 10.1017/ice.2025.10228

**Published:** 2026-01

**Authors:** Charlie Tan, Amrita Bharat, Erin McGill, Robyn Mitchell, Olivia Varsaneux, Kristine Cannon, Marthe K. Charles, Jeannette L. Comeau, Ian Davis, Johan Delport, Tanis C. Dingle, Philippe J. Dufresne, Chelsey Ellis, Jennifer Ellison, Amna Faheem, Charles Frenette, Linda Hoang, Susy Hota, Kevin Katz, Pamela Kibsey, Julianne Kus, Bonita Lee, Xena Li, Yves Longtin, Kathy Malejczyk, Shazia Masud, Dominik Mertz, Sonja Musto, Kishori Naik, Senthuri Paramalingam, Susan M. Poutanen, Dale Purych, Stephanie W Smith, Jocelyn A Srigley, Reena Titoria, Jen Tomlinson, Xuetao Wang, Titus Wong, Deborah Yamamura, Allison McGeer

**Affiliations:** 1 Department of Medicine, Unity Health Toronto, Toronto, ON, Canada; 2 Antimicrobial Resistance and Nosocomial Infections, National Microbiology Laboratory, Public Health Agency of Canada, Winnipeg, MB, Canada; 3 Canadian Nosocomial Infection Surveillance Program, Public Health Agency of Canada, Ottawa, ON, Canada; 4 Infection Prevention and Control, Alberta Health Services, Calgary, AB, Canada; 5 Medical Microbiology and Infection Prevention and Control, Vancouver Coastal Health, Vancouver, BC, Canada; 6 Department of Pediatrics, Dalhousie University, Halifax, NS, Canada; 7 Department of Medicine, Dalhousie University, Halifax, NS, Canada; 8 Department Medical Microbiology, Schulich School of Medicine & Dentistry, Western University, London, ON, Canada; 9 Department of Pathology and Laboratory Medicine, University of Calgary, Calgary, AB, Canada; 10 Laboratoire de Santé Publique du Québec, Institut National de Santé Publique du Québec, Sainte-Anne-de-Bellevue, QC, Canada; 11 Department of Microbiology, The Moncton Hospital, Moncton, NB, Canada; 12 Infection Prevention and Control, North York General Hospital, Toronto, ON, Canada; 13 Department of Medicine, McGill University Health Centre, Montréal, QC, Canada; 14 BC Centre for Disease Control, Vancouver, BC, Canada; 15 Department of Medicine, University Health Network, Toronto, ON, Canada; 16 Shared Hospital Laboratory, Toronto, ON, Canada; 17 Laboratory Medicine and Molecular Diagnostics, Sunnybrook Health Sciences Centre, Toronto, ON, Canada; 18 Department of Microbiology, Vancouver Island Health Authority, Victoria, BC, Canada; 19 Public Health Ontario Laboratory, Toronto, ON, Canada; 20 Department of Pediatrics, Stollery Children’s Hospital, University of Alberta, Edmonton, AB, Canada; 21 Department of Medicine, Jewish General Hospital, Montréal, QC, Canada; 22 Department of Medical Microbiology, Saskatchewan Health Authority, Regina, SK, Canada; 23 Department of Pathology and Laboratory Medicine, Surrey Memorial Hospital, Fraser Health, Surrey, BC, Canada; 24 Departments of Medicine, Clinical Epidemiology and Biostatistics, Pathology and Molecular Medicine, and Michael G. DeGroote Institute for Infectious Diseases Research, McMaster University, Hamilton, ON, Canada; 25 Infection Prevention and Control, Health Sciences Centre, Winnipeg, MB, Canada; 26 West Park Healthcare Centre, Toronto, ON, Canada; 27 Scarborough Health Network, Toronto, ON, Canada; 28 Department of Microbiology, University Health Network/Sinai Health, Toronto, ON, Canada; 29 Faculty of Medicine, University of Alberta, Edmonton, AB, Canada; 30 Department of Pathology and Laboratory Medicine, BC Women’s Hospital and BC Children’s Hospital, Vancouver, BC, Canada; 31 Infection Prevention and Control, Provincial Health Services Authority, Burnaby, BC, Canada; 32 Infection Prevention and Control, Fraser Health, Surrey, BC, Canada; 33 Department of Pathology and Molecular Medicine, McMaster University, Hamilton, ON, Canada

## Abstract

**Objective:**

To assess preparedness for *Candida auris* in Canadian hospitals.

**Design:**

Cross-sectional survey.

**Setting:**

Canadian Nosocomial Infection Surveillance Program (CNISP) hospitals.

**Methods:**

In June 2024, surveys were e-mailed to the infection prevention and control departments of 109 CNISP hospitals and their 33 microbiology laboratories. The surveys assessed policies for patient screening/management and laboratory processes supporting *C. auris* transmission prevention. Results were compared to a similar 2018 survey.

**Results:**

All 109 hospitals and 32/33 laboratories responded. Most hospitals had policies for admission screening (80%, 87/109) and policies/defined plans for post-exposure screening (95%, 104/109). Policy presence increased from 18% to 73% in 56 hospitals completing both 2018 and 2024 surveys (*P* < 0.001). Among hospitals with admission screening policies, 69% (60/87) screened for recent out-of-country hospitalization. All but one hospital implemented transmission-based precautions for cases; 70% (76/109) continued precautions indefinitely. Overall, 94% (99/105; excluding hospitals with exclusively private rooms) and 55% (60/109) of hospitals screened roommates and wardmates, respectively. Frequency and timing of screening and policies regarding precautions for exposed patients varied. All hospitals used axilla and groin swabs, at minimum, for screening. Most (81%, 26/32) laboratories identified all clinically significant *Candida* isolates to species level, increasing from 48% to 85% (*P* < 0.001) in the 27 laboratories completing both 2018 and 2024 surveys. Twenty-four laboratories (75%) had standard operating procedures for processing screening specimens; 96% (23/24) used direct plating onto chromogenic agar.

**Conclusions:**

Despite progress in *C. auris* preparedness, areas for improvement remain. Variability in practice may be related to evidence gaps and resource constraints.

## Introduction

*Candida auris* is an emerging fungus that is highly transmissible in healthcare settings and frequently multidrug-resistant.^1–3^ Healthcare-associated outbreaks with *C. auris* are increasingly reported, and invasive infections are associated with substantial mortality.^2,3^ The World Health Organization, Centers for Disease Control and Prevention, and Public Health Agency of Canada have identified *C. auris* as a critical and urgent threat.^4–6^ As emphasized in several recent national guidelines, effective infection prevention and control (IPAC) practices and laboratory capacity for surveillance, identification and management of colonized/infected patients are vital to prevent transmission in healthcare facilities.^7–9^

In Canada, *C. auris* was first identified in 2012. The first multidrug-resistant case was reported in 2017, and hospital transmission was first reported in 2018.^10–12^ In 2018, the Canadian Nosocomial Infection Surveillance Program (CNISP), a national collaboration between the Public Health Agency of Canada and the Association of Medical Microbiology and Infectious Diseases Canada that represents approximately 37% of acute care hospital beds in the country, surveyed member hospitals to assess preparedness for *C. auris*, and identified significant gaps in both IPAC and microbiology laboratory capacity.^13,14^ As of September 2024, a total of 62 patients colonized/infected with *C. auris* have been identified in Canada, with evidence of clonal transmission within hospitals.^12,15^ Given the increasing recognition of *C. auris* in Canadian hospitals, CNISP hospitals were re-surveyed in 2024 to evaluate progress in *C. auris* preparedness.

## Methods

The 2018 surveys were updated, resulting in 14 IPAC and 15 microbiology laboratory questions (see **Supplementary Material**). The surveys were emailed to the 109 CNISP hospitals and the 33 microbiology laboratories that serve them in June 2024, with a link via LimeSurvey (https://www.limesurvey.org/), followed by three reminders. Responses were described using medians with interquartile ranges for continuous variables and counts with percentages for categorical variables. Responses were compared between pediatric specialty and adult/mixed hospitals, across geographic regions, and between hospitals with and without identified cases of *C. auris,* using Chi-square or Fisher’s exact tests as appropriate. Among hospitals and laboratories completing both the 2018 and 2024 surveys, responses from the two surveys were compared using McNemar’s test. Data were analyzed using Microsoft Excel (Redmond, WA) and R version 4.4.2 (Vienna, Austria).

## Results

Completed IPAC surveys were received from all 109 CNISP hospitals, 12 (11%) of which were pediatric specialty hospitals. With regard to geographic distribution, 40% (44/109) were in Western Canada (British Columbia, Alberta, Saskatchewan, Manitoba), 36% (39/109) were in Central Canada (Ontario, Québec, Nunavut), and 24% (26/109) were in Eastern Canada (New Brunswick, Nova Scotia, Prince Edward Island, Newfoundland, and Labrador). Median hospital bed size was 178 beds (range <5 to 1105). Most hospitals have not encountered a patient colonized/infected with *C. auris*: between January 1, 2012 and September 30, 2024, 27 patients (median 1 per hospital, range 1 to 4) colonized/infected with *C. auris* had been identified in 17 (16%) CNISP hospitals. Information on receipt of healthcare abroad was available for 16 of the 19 cases identified after January 2019, when active surveillance for *C. auris* was implemented across CNISP hospitals. Of these cases, 11 (69%) reported recent hospitalization outside of Canada.

Overall, 80% (87/109) of CNISP hospitals had a policy for screening for *C. auris* colonization at hospital admission. The majority (69%, 60/87) recommended screening for patients recently (eg, in the past 12 months) hospitalized outside of Canada. Among these, 82% (49/60) recommended screening of additional patient groups (Table 1). Twenty-five hospitals (29%, 25/87) were more restricted in admission screening, most commonly recommending screening for patients with recent hospitalization outside of Canada who were also colonized with carbapenemase-producing Enterobacterales. All but two hospitals that did not screen for out-of-country hospitalization reported they would do so if there were no resource limitations, and 12 hospitals (11%, 12/109) indicated they would screen all admissions if they had sufficient resources.

**Table 1.** Policies related to patient screening to detect *Candida auris* colonization in Canadian Nosocomial Infection Surveillance Program hospitals, 2024

Policies, N (%)	Hospitals (n = 109), N (%)
Recommended some screening for *C. auris* colonization	
At admission	87 (80)
For patients exposed during admission^a^	103 (94)
Patients recommended for screening at admission	
Recent (eg, within 12 mo) hospitalization outside of Canada	60 (55)
Only criterion considered	11 (10)
Additional criteria^b^	
Recent (eg, within 12 mo) out-of-province hospitalization	28 (26)
Known previous colonization/infection with *C. auris*	25 (23)
Transfer from healthcare facility with known *C. auris* transmission	11 (10)
Known exposure to patient with *C. auris* colonization/infection	8 (7)
Other^c^	6 (6)
Recent hospitalization outside of Canada **and** colonization with CPE	18 (17)
Only criterion considered	15 (14)
Additional criteria^d^	
Known previous colonization/infection with *C. auris*	3 (3)
Transfer from healthcare facility with known *C. auris* transmission	3 (3)
Known previous colonization/infection with *C. auris* alone	7 (6)
Former roommate of patient colonized/infected with *C. auris*	81 (74)
Always	27 (25)
If follow-up screening not completed during previous admission	54 (50)
Patients recommended for screening following exposure during admission	
Roommates of patients colonized/infected with *C. auris* ^e,^ ^f^	99 (94)
Wardmates of patients colonized/infected with *C. auris* ^g,^ ^h^	60 (55)
Exposure to room potentially contaminated by patient colonized/infected with *C. auris* ^i^	20 (18)
Exposure to equipment potentially contaminated by patient colonized/infected with *C. auris* ^i^	5 (5)
Other^j^	6 (6)
Timing and frequency of roommate screening^k^	
Single test	21 (21)
Two tests	10 (10)
Weekly	5 (5)
Days 0 and 14	4 (4)
Two tests in one week	1 (1)
Three tests	50 (51)
Weekly	32 (32)
Days 0, 7 and 21	15 (15)
Each test separated by at least 24 h	2 (2)
Days 0, 3 and 7	1 (1)
Four tests, weekly	8 (8)
Weekly	10 (10)
Until hospital discharge of roommate	7 (7)
On day 14, then weekly until hospital discharge of roommate	3 (3)
Timing and frequency of wardmate screening^l^	
Single test	12 (20)
Two tests, weekly	5 (8)
Three tests, weekly	9 (15)
Four tests, weekly	2 (3)
Weekly	25 (42)
Until hospital discharge of index case	13 (22)
Until hospital discharge of wardmate	7 (12)
Until no further transmission	5 (8)
Situation specific	7 (12)

Abbreviations: CPE, carbapenemase-producing Enterobacterales.

a Of the 103 hospitals that recommended screening for patients exposed during admission, 93 had a policy and 10 did not have a policy but described a specific plan for screening. 1 hospital had a policy but did not recommend post-exposure screening, and was therefore not included.

b Of the 49 hospitals that recommended screening for additional criteria, 29 recommended screening for 1 additional criterion, 9 for 2 additional criteria, and 11 for 3 additional criteria. Sum of additional criteria considered is therefore greater than 49.

c Other included colonization/infection with CPE (4), admission to intensive care unit (1), receipt of healthcare not requiring hospitalization (eg, hemodialysis, day surgery) outside of Canada (1).

d All 3 hospitals that recommended screening for additional criteria performed screening for both criteria listed. Sum of additional criteria considered is therefore greater than 3.

e Denominator of 105, excluding 4 hospitals that had exclusively private rooms.

f Of the 99 hospitals that screened roommates, 89 had a policy and 10 did not have a policy but described a specific plan for screening.

g Of the 60 hospitals that screened wardmates, 51 had a policy and 9 did not have a policy but described a specific plan for screening.

h 7 hospitals only screened wardmates in rooms directly beside and across hall from index case.

i Numbers may be underestimated as these options were not listed specifically on the survey, but written in by respondents as “Other”.

j Other included point prevalence surveys in high-risk units (eg, intensive care unit, hematology-oncology unit) (4), at discretion of infection prevention and control (2).

k Denominator of 99, excluding 6 hospitals that did not screen roommates and 4 hospitals with only private rooms.

l Denominator of 60, excluding 49 hospitals that did not screen wardmates.

Most (86%, 94/109) hospitals had a policy for screening patients exposed to *C. auris* during hospitalization, one of whom did not recommend doing so. An additional 9% (10/109) reported not having a formal policy, but having specific plans for screening in the event that exposure occurred. Cumulatively, 94% (99/105: excluding four hospitals with only private rooms) of hospitals recommended screening roommates and 55% (60/109) recommended screening wardmates. Hospital policies varied widely in the number and frequency of follow-up screening tests recommended for both roommates and wardmates (Table 1). Most hospitals (61%, 64/105; excluding four hospitals with only private rooms) recommended roommates be managed with transmission-based precautions until at least two follow-up screens were negative or until hospital discharge (Table 2). Overall, among 56 hospitals completing both 2018 and 2024 surveys, the presence of any screening policy increased from 18% to 73% (*P* < 0.001).

**Table 2.** Policies related to transmission-based precautions for patients colonized/infected with or exposed to *Candida auris* in Canadian Nosocomial Infection Surveillance Program hospitals, 2024

Policies, N (%)	Hospitals (n = 109), N (%)
Components of transmission-based precautions	
Private room	108 (99)
Gowns and gloves	108 (99)
Enhanced environmental cleaning/disinfection	108 (99)
Increase from once daily to twice daily room cleaning/disinfection^a^	87 (80)
Curtain change after hospital discharge	75 (69)
Enhanced terminal clean after hospital discharge^b^	25 (23)
Dedicated washroom	101 (93)
Dedicated mobile equipment	98 (90)
Dedicated healthcare staff	15 (14)
Duration of precautions for patients colonized/infected with *C. auris*	
No precautions	1 (1)
Not yet determined and/or situation-specific	14 (13)
Continued throughout admission and re-implemented upon future readmission	76 (70)
Continued throughout admission but not re-implemented upon future readmission unless screen at readmission positive	5 (5)
Continued until specific number of screening swabs are negative^c^	15 (14)
Duration of precautions for roommates^d^	
No precautions	4 (4)
Not yet determined	10 (10)
Until 1 set of screening swabs negative	27 (26)
Until 2 or more sets of screening swabs negative	62 (59)
Until hospital discharge	2 (2)

a In addition to twice daily room cleaning/disinfection, 7 hospitals changed from alcohol-based hand rub to sporicidal wipes and soap/water, and 1 hospital added steam cleaning of drains.

b Among hospitals that specified components of enhanced terminal clean, 9 used hydrogen peroxide fogging or ultraviolet light disinfection, 3 performed double cleaning/disinfection, 3 performed triple cleaning/disinfection, 1 required environmental services supervisor inspection after terminal clean, and 1 performed environmental sampling before room was re-opened.

c 1 negative set (2), 2 negative sets (5), 3 negative sets (8).

d Denominator of 105, excluding 4 hospitals that only had private rooms.

All but one small, rural hospital (<30 beds) reported recommending transmission-based precautions for patients colonized with *C. auris*, including private room, donning of gowns and gloves, and enhanced cleaning/disinfection. Dedicated washroom and dedicated mobile equipment were required by 93% (101/109) and 90% (98/109) of hospitals, respectively. Either accelerated hydrogen peroxide or sodium hypochlorite-based products were used for environmental disinfection by 94% (103/109) of hospitals. For *C. auris* colonized/infected patients, 70% (76/109) of hospitals recommended continuing transmission-based precautions indefinitely (Table 2).

Pediatric hospitals were less likely than adult/mixed hospitals to perform any screening at admission (33% (4/12) vs 86% (83/97), *P* < 0.001), to screen at admission for recent out-of-country hospitalization (25% (3/12) vs 59% (57/97), *P* = 0.03), to screen wardmates (33% (4/12) vs 58% (56/97), *P* = 0.04), to continue transmission-based precautions indefinitely for patients colonized/infected with *C. auris* (42% (5/12) vs 73% (71/97), *P* = 0.04), and to continue precautions for roommates until one or more screening swabs were negative or until hospital discharge (55% (5/9) vs 90% (86/96), *P* = 0.04) (Supplementary Table 1). Hospitals in Western Canada were less likely to screen wardmates than hospitals in Central and Eastern Canada (32% (14/44) vs 82% (32/39) and 54% (14/26), respectively, *P* < 0.001). They were also less likely to screen at admission for recent out-of-country hospitalization (23% (10/44) vs 69% (27/39) and 88% (23/26), respectively, *P* < 0.001). Hospitals in Western and Central Canada were more likely to collect multiple sets of post-exposure screening swabs for roommates compared to hospitals in Eastern Canada, the majority of which collected a single set (95% (40/42) and 94% (32/34), respectively vs 26% (6/23), *P* < 0.001). Hospitals in Central Canada were more likely to require multiple sets of negative screening swabs to discontinue precautions for roommates than hospitals in Western and Eastern Canada (95% (35/37) vs 67% (29/43) and 56% (14/25), respectively, *P* < 0.001) (Supplementary Table 2). Hospitals with previously identified cases of *C. auris* were more likely than hospitals without previous cases to continue post-exposure screening for roommates until discharge (29% (5/17) vs 6% (5/82), *P* = 0.02) and to collect multiple sets of post-exposure screening swabs for roommates (94% (16/17) vs 76% (62/82), p = 0.02) (Supplementary Table 3).

Of the 83% (90/109) of hospitals that provided data on specimen types recommended for screening, 44% (40/90) recommended pooled axilla and groin swabs, 40% (36/90) recommended separate axilla and groin swabs, and 16% (14/90) recommended pooled axilla, groin and nares swabs. Among these hospitals, 29% (26/90), 17% (15/90), 16% (14/90), and 8% (7/90) also recommended screening wounds and exit sites (if present), urine, nares and rectum, respectively.

Microbiology laboratory surveys were completed by 97% (32/33) of CNISP laboratories. The results of the microbiology survey are summarized in Table 3. All clinically significant *Candida* isolates were identified to the species level by 81% (26/32) of laboratories; this increased from 48% to 85% (*P* < 0.001) in the 27 laboratories that completed both 2018 and 2024 surveys. The remaining laboratories identified isolates to the species level for at least all sterile sites (16%, 5/32) or all blood cultures (3%, 1/32).

**Table 3.** Laboratory practices related to *Candida auris* in microbiology laboratories serving Canadian Nosocomial Infection Surveillance Program hospitals, 2024

Practices, N (%)	Hospitals (n = 32), N (%)
Types of *Candida* isolates identified to species level	
All clinically significant^a^ *Candida* isolates	26 (81)
*Candida* isolates from all sterile sites^b^	5 (16)
*Candida* isolates from blood cultures only	1 (3)
*Candida* isolates selected for antifungal susceptibility testing	
None	1 (3)
All isolates from blood and CSF cultures	26 (81)
Only some isolates from blood and CSF cultures^c^	5 (16)
All isolates from other (ie, non-blood, non-CSF) sterile sites	17 (53)
Only some isolates from other sterile sites^d^	13 (41)
Some isolates from non-sterile sites^e^	19 (59)
*Candida auris* isolated from screening specimens	15 (47)
Antifungal susceptibility testing	
Performed in-house^f^	18 (56)
Sensititre^TM^ YeastOne^TM^ Y09	11 (34)
Gradient strip	4 (13)
VITEK^®^ 2 YS08	4 (13)
Broth microdilution (according to CLSI M27)	1 (3)
Referred to other laboratory^g^	20 (63)
Antifungal agents for which susceptibility testing routinely performed	
Fluconazole	30 (94)
At least 1 azole other than fluconazole	28 (88)
At least 1 echinocandin (anidulafungin, caspofungin, micafungin)	30 (94)
Amphotericin B	26 (81)
5-flucytosine	16 (50)
Procedures for processing screening swabs for *Candida auris* (among 24 laboratories with standard operating procedures for in-house processing)	
Initial processing of swabs	
Direct plating onto chromogenic agar	23 (96)
Micronostyx COLOREX^TM^/CHROMagar^TM^ Candida Plus	13 (54)
BD BBL^TM^ CHROMagar^TM^ Candida	4 (17)
Bio-Rad CandiSelect^TM^	4 (17)
Thermo Fisher Brilliance^TM^ Candida	2 (8)
Direct plating onto Sabouraud agar	1 (4)
Temperature at which plates are incubated	
35ºC to 37ºC	14 (58)
40ºC to 42ºC	10 (42)
Duration of incubation before specimen called negative	
24 h	2 (8)
48 h	9 (38)
3 d to 5 d	4 (17)
7 d	9 (38)

a Clinically significant isolates are those for which laboratory standard operating procedures would require isolate species being reported as present.

b Of the 5 hospitals that identified *Candida* isolates from all sterile sites to species level, 2 also did so for isolates from some non-sterile sites: 1 on request and 1 for all *C. auris* isolates or on request.

c Included all non-*albicans* species (4), persistent candidemia/suspected treatment failure (1), all *C. glabrata* isolates (1), on request (1).

d Included all non-*albicans* species (6), on request (4), persistent infection/suspected treatment failure (2), all *C. auris* isolates (2), all biopsy specimens (1), prosthetic joints (1), brain (1), intraabdominal (1), depending on *Candida* species (1).

e Included on request (13), all *C. auris* isolates (2), suprapubic urine (1), ureteric stents (1), deep wounds (1), vaginal (1), vaginal with treatment failure only (1), depending on *Candida* species (1).

f 1 laboratory used both Sensititre^TM^ YeastOne^TM^ Y09 and gradient strips, and 1 laboratory used both VITEK^®^ 2 YS08 and gradient strips. Sum of laboratories is therefore greater than 18.

g 6 laboratories performed both in-house susceptibility testing and referred to other laboratories. Sum of laboratories is therefore greater than 32.

Susceptibility testing for at least some *Candida* isolates was performed by 97% (31/32) of laboratories, with 53% (17/32) having in-house capacity. Most laboratories (75%, 24/32) processed screening swabs in-house and had a standard operating procedure to identify *C. auris* colonization, while 3% (1/32) sent screening specimens to a referral laboratory. In the 27 laboratories responding to both 2018 and 2024 surveys, the number that reported having procedures to process screening specimens to detect *C. auris* increased from 11% to 81% (*P* = 0.003). Of the 24 laboratories that processed screening swabs in-house, ESwabs^TM^ and Opti-Swabs^®^ were the most commonly used swabs (75%, 18/24), and Liquid Amies was the most commonly used transport medium (58%, 14/24). The majority (96%, 23/24) of laboratories plated swabs directly onto chromogenic agar; one (4%) used Sabouraud agar. No laboratories used dulcitol broth enrichment or polymerase chain reaction (PCR)-based methods for detection. Plates were incubated for 24 hours to 7 days, with 54% (13/24) of laboratories requiring incubation periods of 72 hours or greater before reporting a screening specimen as negative.

## Discussion

Our results demonstrate that since 2018, CNISP hospitals and their microbiology laboratories have made substantial progress in preparedness for *C. auris*. In 2018, only 18% of hospitals had a policy for *C. auris* screening, and 63% did not recommend patient screening;^14^ by 2024, 80% reported having a policy for admission screening and 86% for post-exposure screening (with an additional 9% not having a policy but having a specific plan for post-exposure screening). Furthermore, the majority of hospitals implemented transmission-based precautions and enhanced environmental disinfection with appropriate agents for cases and exposed roommates.

In the Canadian federation, health is a provincial/territorial responsibility. To date, only three provinces have designated *C. auris* a reportable pathogen (one each in 2021, 2024 and 2025).^16–18^ Nonetheless, provincial/territorial public health laboratories have been requesting all *C. auris* isolates to be submitted to them for at least the past decade. Furthermore, many laboratories refer isolates of all *Candida* species requiring susceptibility testing to their provincial/territorial public health laboratories, and for this project, we asked CNISP hospitals to report all patients identified as colonized/infected with *C. auris*, including before CNISP surveillance started in 2019. This information suggests that *C. auris* remains uncommon in Canada, and that the 62 cases reported since 2012 represent a majority of identified cases, although it is very likely that some colonized cases were not identified due to lack of screening.

Where data are available, most cases have occurred in persons with out-of-country healthcare exposure. Based on similar data in other jurisdictions, national guidance in the United States, the United Kingdom and Canada all recommend *C. auris* screening for patients who have been hospitalized in other countries.^7–9^ Therefore, the 45% of Canadian hospitals that currently do not recommend screening for out-of-country hospitalization alone or have more restricted screening criteria may miss introductions of *C. auris*. For Canadian hospitals, this is particularly important given the dramatic increase in *C. auris* colonization and infection that has been observed in the United States.^19^

The purpose of the CNISP *C. auris* working group is to provide surveillance data for *C. auris* in Canadian hospitals, and to share information to support an early and effective response to this emerging pathogen, with the aim of preventing progression to endemicity. However, the variability in screening practices and duration of transmission-based precautions used for colonized/infected patients identified in our survey highlights the absence of evidence to inform policy. Although national and provincial guidance have been developed, much of this is based on expert opinion.^7–9^ Building an evidence base to support transmission control programs is therefore both essential and urgent. The recent United States Department of Veterans Affairs research agenda for prevention of transmission of antimicrobial resistant organisms in hospitals highlights the evidence gaps that need to be addressed.^20^ One critical question is how and when to expand admission screening as the epidemiology of *C. auris* changes. Studies for other antimicrobial resistant organisms (such as carbapenemase-producing Enterobacterales) suggest that expanding admission screening may be cost-effective even at prevalence rates well below 1%, although budgetary pressures on microbiology laboratories make expansion uniformly challenging.^21,22^ Additional evidence to support decision-making about epidemiologic thresholds to increase screening for *C. auris* colonization are urgently needed. Other important questions include the frequency and duration of contact screening, criteria for discontinuing transmission-based precautions in exposed and colonized patients, how to balance the negative consequences of precautions and patient/family preferences against the benefits of reduced transmission, and strategies to incorporate the environmental costs of precautions into decisions about transmission prevention.

Laboratory capacity for *Candida* species identification and screening to detect *C. auris* colonization were identified as challenges in the 2018 version of this survey. Substantial gains have been made: 81% of CNISP microbiology laboratories identified all clinically significant *Candida* isolates to species level, and 75% process screening swabs to detect *C. auris* colonization in-house, compared to 48% and 11% in 2018, respectively.^14^ Improved identification of *C. auris* is primarily the result of widespread adoption of matrix-assisted laser desorption/ionization-time of flight (MALDI-TOF) mass spectrometry for organism identification, now employed by all 32 responding CNISP microbiology laboratories, as well as improvements to prior limitations in *C. auris* spectra in MALDI-TOF databases.

The improved ability of laboratories to process screening swabs for *C. auris* colonization is valuable progress. However, all laboratories used direct plating and culture (ie, no broth enrichment or PCR-based methods), and 6% incubated specimens for only 24 hours, raising the possibility that cases of *C. auris* may be missed.^21^ The limited sensitivity of screening and prolonged incubation times required to properly process screening swabs remain significant challenges, with some guidelines now recommending PCR-based methods.^8,23^ Data evaluating different laboratory approaches to detect *C. auris* colonization are an immediate priority. In addition, increased availability of external quality assessment programs for both culture and PCR-based methods for *C. auris* detection are needed to evaluate current laboratory practices for *C. auris* screening and to support the adoption of best practices as evidence evolves.^24,25^

This survey has limitations. Although it was pilot tested, misinterpretation of questions may have occurred. The generalizability of the practices described may also be limited. Although CNISP member hospitals include approximately 37% of all hospital beds in Canada,^13,26^ with representation in 11 of 13 Canadian provinces and territories, larger academic hospitals are overrepresented, comprising more than half of participating hospitals. CNISP member hospitals may also have greater existing resources and capacity for IPAC than other hospitals.

In conclusion, CNISP hospitals and their microbiology laboratories have significantly improved capacity to detect and control transmission of *C. auris* since 2018. However, gaps remain, and continued efforts to identify risk factors for acquisition and best practices to prevent transmission of this emergent pathogen should be prioritized. Mandatory reporting in all provinces and territories, provincial/territorial and federal collaboration in providing continuing updates and disseminating evidence related to *C. auris* transmission control, research funding to fill gaps in knowledge, and standardized quality assessments of laboratory processes would all help to support the goal of preventing endemicity and optimizing patient care.

## Supporting information

Tan et al. supplementary material Tan et al. supplementary material

## Supplementary material

The supplementary material for this article can be found at https://doi.org/10.1017/ice.2025.10228
