# Supplementary material for: Preparedness for Candida auris in Canadian Nosocomial Infection Surveillance Program (CNISP) hospitals, 2024
Source: Infect Control Hosp Epidemiol. 2025 Oct 30;47(1):39–45. doi: 10.1017/ice.2025.10228 (PMC12780836; doi:10.1017/ice.2025.10228)
Supplement: Tan et al. supplementary material [file S0899823X25102286sup001.docx]

**Supplementary material:**

Preparedness for *Candida auris* in Canadian Nosocomial Infection Surveillance Program (CNISP) hospitals, 2024

Tan C, et al.

**Contents:**

Supplementary Table 1…………………………………….…………………………pg 2

Supplementary Table 2…………………………………….…………………………pg 3

Supplementary Table 2…………………………………….…………………………pg 4

Word versions of surveys to IPAC teams and microbiology laboratories……….pg 5

**Supplementary Table 1:** Policies related to *Candida auris* screening and transmission-based precautions in Canadian Nosocomial Infection Surveillance Program hospitals, 2024, stratified by pediatric specialty versus adult/mixed hospitals

| **Policy, N (%)** | **Pediatric (N=12)** | **Adult/mixed (N=97)** | **p-value** |
| --- | --- | --- | --- |
| Screening for *C. auris* |  |  |  |
| At admission | 4 (33) | 83 (86) | <0.001 |
| For patients exposed during admission | 10 (83) | 94 (97) | 0.09 |
| Admission screening for patients with recent out-of-country hospitalization | 3 (25) | 57 (59) | 0.03 |
| Patients recommended for screening following exposure |  |  |  |
| Roommates of patients colonized/infected with *C. auris*^a^ | 7 (78) | 92 (96) | 0.08 |
| Wardmates of patients colonized/infected with *C. auris* | 4 (33) | 56 (58)^b^ | 0.04 |
| Recommended roommate screening^c^ |  |  | 0.22 |
| 1 test | 2 (29) | 19 (21) |  |
| 2 tests | 2 (29) | 8 (9) |  |
| 3 tests | 2 (29) | 48 (52) |  |
| 4 tests | 1 (14) | 7 (8) |  |
| Until hospital discharge | 0 (0) | 10 (11) |  |
| Precautions recommended indefinitely for patients colonized/infected with *C. auris* | 5 (42) | 71 (73) | 0.04 |
| Recommended duration of precautions for roommates^a^ |  |  | 0.04 |
| No precautions | 2 (22) | 2 (2) |  |
| Not yet determined | 2 (22) | 8 (8) |  |
| Until 1 set of screening swabs negative | 1 (11) | 26 (27) |  |
| Until ≥2 sets of screening swabs negative | 4 (44) | 58 (60) |  |
| Until hospital discharge | 0 (0) | 2 (2) |  |

^a^ Excluding hospitals that only had single rooms. Denominators for pediatric specialty and adult/mixed hospitals are 9 and 96, respectively.

^b^ 7 adult/mixed hospitals only screen wardmates in rooms directly bedside and across hall from index case

^c^ Excluding hospitals that did not perform screening of roommates or only had single rooms. Denominators for pediatric specialty and adult/mixed hospitals are 7 and 92, respectively.

**Supplementary Table 2:** Policies related to *Candida auris* screening and transmission-based precautions in Canadian Nosocomial Infection Surveillance Program hospitals, 2024, stratified by geographic region

| **Policy, N (%)** | **Western (N=44)** | **Central (N=39)** | **Eastern (N=26)** | **p-value** |
| --- | --- | --- | --- | --- |
| Recommend screening for *C. auris* |  |  |  |  |
| At admission | 37 (84) | 27 (69) | 23 (88) | 0.11 |
| For patients exposed during admission | 44 (100) | 36 (92) | 24 (92) | 0.11 |
| Recommend admission screening for patients with recent out-of-country hospitalization | 10 (23) | 27 (69) | 23 (88) | <0.001 |
| Patients recommended for screening following exposure |  |  |  |  |
| Roommates of patients colonized/infected with *C. auris*^a^ | 42 (98) | 34 (92) | 23 (92) | 0.50 |
| Wardmates of patients colonized/infected with *C. auris* | 14 (32)^b^ | 32 (82) | 14 (54) | <0.001 |
| Recommended roommate screening^c^ |  |  |  | <0.001 |
| 1 test | 2 (5) | 2 (6) | 17 (74) |  |
| 2 tests | 6 (14) | 3 (9) | 1 (4) |  |
| 3 tests | 27 (64) | 23 (68) | 0 (0) |  |
| 4 tests | 0 (0) | 3 (9) | 5 (22) |  |
| Until hospital discharge | 7 (17) | 3 (9) | 0 (0) |  |
| Precautions recommended indefinitely for patients colonized/infected with *C. auris* | 32 (73) | 26 (67) | 18 (69) | 0.83 |
| Recommended duration of precautions for roommates^a^ |  |  |  | <0.001 |
| No precautions | 1 (2) | 3 (8) | 0 (0) |  |
| Not yet determined | 2 (5) | 0 (0) | 8 (32) |  |
| Until 1 set of screening swabs negative | 14 (33) | 2 (5) | 11 (44) |  |
| Until ≥2 sets of screening swabs negative | 24 (56) | 32 (86) | 6 (24) |  |
| Until hospital discharge | 2 (5) | 0 (0) | 0 (0) |  |

^a^ Excluding hospitals that only had single rooms. Denominators for Western, Central and Eastern regions are 43, 37 and 25, respectively.

^b^ 7 hospitals in Western Canada only screened wardmates in rooms directly bedside and across hall from index case

^c^ Excluding hospitals that did not perform screening of roommates or only had single rooms. Denominators for Western, Central and Eastern regions are 42, 34 and 23, respectively.

**Supplementary Table 3:** Policies related to *Candida auris* screening and transmission-based precautions in Canadian Nosocomial Infection Surveillance Program hospitals, 2024, stratified by whether hospital has had previous cases of *Candida auris*

| **Policy, N (%)** | **Previous *C. auris* case**  **(n=17)** | **No previous *C. auris* case**  **(n=92)** | **p-value** |
| --- | --- | --- | --- |
| Screening for *C. auris* |  |  |  |
| At admission | 15 (88) | 72 (78) | 0.52 |
| For patients exposed during admission | 17 (100) | 87 (95) | 1 |
| Admission screening for patients with recent out-of-country hospitalization | 6 (35) | 54 (59) | 0.08 |
| Patients recommended for screening following exposure |  |  |  |
| Roommates of patients colonized/infected with *C. auris*^a^ | 17 (100) | 82 (93) | 0.59 |
| Wardmates of patients colonized/infected with *C. auris* | 12 (71)^b^ | 48 (52)^c^ | 0.33 |
| Recommended roommate screening^d^ |  |  | 0.02 |
| 1 test | 1 (6) | 20 (24) |  |
| 2 tests | 3 (18) | 7 (9) |  |
| 3 tests | 8 (47) | 42 (51) |  |
| 4 tests | 0 (0) | 8 (10) |  |
| Until hospital discharge | 5 (29) | 5 (6) |  |
| Precautions recommended indefinitely for patients colonized/infected with *C. auris* | 14 (82) | 62 (67) | 0.22 |
| Recommended duration of precautions for roommates^a^ |  |  | 0.56 |
| No precautions | 0 (0) | 4 (5) |  |
| Not yet determined | 0 (0) | 10 (11) |  |
| Until 1 set of screening swabs negative | 6 (35) | 21 (24) |  |
| Until ≥2 sets of screening swabs negative | 11 (65) | 51 (58) |  |
| Until hospital discharge | 0 (0) | 2 (2) |  |

^a^ Excluding hospitals that only had single rooms. Denominators for hospitals with and without previous *C. auris* cases are 17 and 88, respectively.

^b^ 4 hospitals with previous cases of *C. auris* only screened wardmates in rooms directly bedside and across hall from index case

^c^ 3 hospitals without previous cases of *C. auris* only screened wardmates in rooms directly bedside and across hall from index case

^d^ Excluding hospitals that did not perform screening of roommates or only had single rooms. Denominators for hospitals with and without previous *C. auris* cases are 17 and 82, respectively.

**Word versions of IPAC and Laboratory Surveys:**

**CNISP *Candida auris* INFECTION PREVENTION AND CONTROL PRACTICES Survey**

**Hospitals included (enter name or CNISP site code):** Click here to enter text.

1. Do you have an infection prevention and control policy that determines which patients will be screened for colonization with *C. auris*?

☐ Yes ☐ No ☐ Not sure

If yes, which patient population(s) are included in the policy?

☐ high risk patients on admission, **please select all that apply**:

☐ **ALL** patients recently hospitalized outside of Canada (e.g. in the past 12 months)

☐ **SOME** patients recently hospitalized outside of Canada (e.g. in the past 12 months) – please specify which (e.g. by country/region: ___________________)

☐ Patients recently hospitalized in other Canadian provinces/territories (e.g. in the past 12 months)

☐ **ALL** patients who are Carbapenemase-Producing Organism (CPO) colonized or infected

☐ Patients who are CPO colonized or infected **AND** have a history of hospitalization in other countries (if only some countries, please specify ____________________)

☐ Patients who are *C. auris* positive

☐ Other, please specify: ___________________

☐ patients during admission, **please select all that apply**:

☐ with exposure to antifungals, please specify: ___________________

☐ roommates of a patient identified as colonized or infected with *C. auris*

☐ wardmates of a patient identified as colonized or infected with *C. auris*

☐ patients who have spent time in a room potentially contaminated by a patient colonized or infected with *C. auris*

☐ other, please specify: ___________________

1. If your policy recommends screening of some patients to identify colonization a) what specimens are collected and b) in which specimens have you identified *C. auris*?

☐ Not applicable, no policy or policy does not recommend screening

| **Specimen type** | **Collected as per policy** | **Identified *C. auris*** |
| --- | --- | --- |
| Groin | ☐ | ☐ |
| Axilla | ☐ | ☐ |
| Pooled groin/axilla | ☐ | ☐ |
| Pooled groin/axilla/nares | ☐ | ☐ |
| Rectal | ☐ | ☐ |
| Nares | ☐ | ☐ |
| Urine | ☐ | ☐ |
| Other, please specify: | ☐ | ☐ |

1. Which specimen type has been most likely to be positive in patients colonized with *C. auris*?

Specify ________________

Or ☐ Not applicable: we have too few positive specimens to assess this

1. If you had no resource limitations, what patient screening would you be recommending for your hospital(s) in 2024? (check as many as applicable)

☐ high risk patients on admission, please select all that apply:

☐ **ALL** patients recently hospitalized outside of Canada (e.g. in the past 12 months)

☐ **SOME** patients recently hospitalized outside of Canada (e.g. in the past 12 months) - please specify which (e.g. by country/region: ___________________)

☐ Patients recently hospitalized in other Canadian provinces/territories (e.g. in the past 12 months)

☐ **ALL** patients who are CPO colonized or infected

☐ Patients who are CPO colonized or infected **AND** have a history of hospitalization in other countries (if only some countries, please specify ____________________)

☐ Other, please specify: ___________________

☐ patients during admission

☐ with exposure to antifungals, please specify: ___________________

☐ roommates of a patient identified as colonized or infected with *C. auris*

☐ wardmates of a patient identified as colonized or infected with *C. auris*

☐ patients who have spent time in a room potentially contaminated by a patient colonized or infected with *C. auris*

☐ other, please specify: ___________________

1. How long does your hospital continue to screen/test roommates of a positive *C. auris* patient (either in practice or according to policy)?

☐ We do not screen/test roommates of a positive *C. auris* patient

☐ Until roommate has one negative test

☐ Until roommate has two negative tests

☐ For 3 weeks (or until discharge if earlier)

☐ For 4 weeks (or until discharge if earlier)

☐ Until discharge, however long it is

☐ other, please specify: ___________________

1. If a roommate of a *C. auris* patient is readmitted at a later date, are they flagged for rescreening?

☐ Yes, always

☐ Yes, if they were discharged before follow up was complete

☐ No

1. How frequently are roommates of a positive *C. auris* patient tested during the above indicated period (either in practice or according to policy)?

☐ N/A – we do not test roommates

☐ Weekly

☐ Twice per week

☐ Biweekly (i.e. every two weeks)

☐ Other, please specify: __________________

1. How long does your hospital continue to screen/test wardmates of a positive *C. auris* patient (either in practice or according to policy)?

☐ We do not screen/test wardmates of a positive *C. auris* patient

☐ Until wardmate has one negative test

☐ Until wardmate has two negative tests

☐ For 3 weeks (or until discharge if earlier)

☐ For 4 weeks (or until discharge if earlier)

☐ Until discharge, however long it is

☐ other, please specify: ___________________

1. How frequently are wardmates of a positive *C. auris* patient tested during the above follow up period (either in practice or according to policy)?

☐ N/A – we do not test wardmates

☐ Weekly

☐ Twice per week

☐ Biweekly (i.e. every two weeks)

☐ Other, please specify: __________________

1. Are additional precautions (e.g. contact precautions) used for roommates with a significant exposure (as defined by your hospital) to a patient colonized or infected with *C. auris* (either in practice or according to policy) pending the results of screening?

☐ No,

☐ Yes, until they have been screened once and found to be negative

☐ Yes, until they have been screened 2 or 3 times and found to be negative

☐ Yes, until discharge

☐ Yes, other, please specify: _________________________________

1. Which components of additional precautions (e.g. contact precautions) does your hospital implement for a *C. auris* positive patient? Please check all that apply.

☐ N/A – we do not implement additional precautions for *C. auris* positive patients

☐ Gowns

☐ Gloves

☐ Enhanced environmental cleaning/disinfection

☐ Dedicated mobile medical equipment

☐ Placement in a single room

☐ Dedicated toileting facilities (i.e. no one shares bathroom)

☐ Other, please specify: __________________

1. If yes to enhanced environmental cleaning, what is enhanced about your cleaning?

☐ Twice per day vs. once per day

☐ Different products for either daily or discharge clean, please specify:

☐ Change curtains after discharge

☐ Different process for daily clean, please specify: ___________________

☐ Different process for post-discharge, please specify: _______________

☐ Other, please specify: __________________________________

**CNISP *Candida auris* LABORATORY PRACTICES Survey**

**Name of laboratory:** ​Click here to enter text.​

**Province or Territory:**  ​{include drop down list of provinces and territories}

1. For which types of *Candida* isolates do you identify or attempt to identify to the species level? Please check all that apply

☐ All clinically significant *Candida* isolates

☐ No Candida isolates –> skip to Q3

☐ *Candida* isolates from blood cultures

☐  *Candida* isolates from CSF cultures

☐ *Candida* isolates from cultures of sterile sites other than blood or CSF

☐ Some isolates from non-sterile sites

If yes, please specify the criteria: Click here to enter text.

1. Which method do you use for the identification of *Candida* to the species level? Please check all that apply

☐ Vitek 2 YST (bioMerieux)

☐ API 20C AUX (bioMerieux)

☐ Phoenix Yeast ID system (BD)

☐ Microscan (Beckman Coulter)

☐ Matrix-assisted laser desorption/ionization time-of-flight (MALDI-TOF)

☐ Biotype (Bruker) – clinical/commercial database

☐ Biotype (Bruker) – RUO/research database

☐ Vitek MS (bioMerieux) clinical/commercial database

☐ Vitek MS (bioMerieux) RUO/research database

☐ Chromogenic agar

☐ PCR/Sequencing, if yes, please specify which isolates have PCR/sequencing, and what type of

sequencing is done : Click here to enter text.

☐ Other (please specify): Click here to enter text.

1. For which types of *Candida* isolates do you perform (or send to reference lab to perform) antifungal susceptibility testing? Please check all that apply

☐ All *Candida* isolates -> skip to Q4

☐ No *Candida* isolates -> skip to Q4

☐ Some *Candida* isolates

Please specify which isolates (please select all that apply)

☐ All isolates from blood/CSF cultures (i.e. at least one isolate per episode of candidemia)

☐ All isolates from cultures of other sterile sites (i.e. other than blood and CSF)

☐ Some isolates from blood cultures, specify criteria: Click here to enter text.

☐ Some isolates from cultures of other sterile sites (i.e. other than blood)

specify criteria: Click here to enter text.

☐ Some isolates from non-sterile sites, specify criteria: Click here to enter text.

☐ *Candida auris* isolated from surveillance/screening specimens

1. How/where is your antifungal susceptibility testing performed? Please check all that apply

☐ In our laboratory

Please specify method(s) used (please check as that apply)

☐ Broth microdilution according to CLSI M27 method

☐ Sensititre YeastOne Y09 Panel (Thermofisher/Oxoid)

☐ Gradient strips (e.g E test)

☐ Disc diffusion (Kirby Bauer) according to CLSI M44

☐ Vitek 2 YS08 cards (bioMérieux)

☐ Other, specify:

☐ Referred to our provincial public health laboratory

☐ Referred to other lab, please specify lab: Click here to enter text.

1. Which antifungal agents are routinely tested (by your lab, or your reference lab)? Please check all that apply

☐ None, we do not do or request susceptibility testing routinely on any *Candida* isolates

Fluconazole ☐ Yes ☐ No ☐ Unknown

Voriconazole ☐ Yes ☐ No ☐ Unknown

Posaconazole ☐ Yes ☐ No ☐ Unknown

Itraconazole ☐ Yes ☐ No ☐ Unknown

Isavuconazole ☐ Yes ☐ No ☐ Unknown

Micafungin ☐ Yes ☐ No ☐ Unknown

Caspofungin ☐ Yes ☐ No ☐ Unknown

Anidulafungin ☐ Yes ☐ No ☐ Unknown

Amphotericin B ☐ Yes ☐ No ☐ Unknown

Flucytosine ☐ Yes ☐ No ☐ Unknown

Other, please specify __________________ ☐ Yes ☐ No ☐ Unknown

1. How do you report susceptibility results? Please check all that apply

☐ Minimum Inhibitory Concentration (MIC) only

☐ Interpretation as per CLSI guidelines where breakpoints are available

☐ Interpretation as per CLSI guidelines using epidemiologic cutoff values

☐ Interpretation as per EUCAST guidelines (using clinical breakpoints or ECOFFs)

☐ Interpretation as per CDC interim *C. auris* breakpoints

☐ Other, specify: Click here to enter text.

1. Do you have a laboratory procedure/SOP for processing screening swabs from patients to detect **colonization** with *C. auris* (e.g. for exposed contacts of a case)?

☐ No (or not yet) -> end of survey

☐ Yes, we would send to our provincial laboratory, which has a procedure -> end of survey

☐ Yes, we have our own policy/procedure - continue to 8a

If replied ‘Yes, we have our own policy/procedure’, please answer the following questions

1. Which specimens do you accept for screening? Please check all that apply

☐ Groin

☐ Axilla

☐ Pooled axilla/groin

☐ Rectal

☐ Nares

☐ Urine

☐ Other, please specify:

1. In your experience, which specimen type is most likely to be positive in patients colonized with *C. auris*?

Specify ________________

Or ☐ Not applicable: we have too few positive specimens to assess this

1. What swabs are used to collect *C. auris* screening specimens? Please check all that apply

☐ Cotton/Dracon/Rayon swabs

☐ E-swabs or Opti-swabs

☐ Other, please specify:

1. What transport media are used? Please check all that apply

☐ Stuart Gel

☐ Liquid Stuart

☐ Amies Charcoal

☐ Amies Clear

☐ Liquid Amies

☐ Amies Plus (light charcoal) Gel

☐ Other, please specify :

1. Do you perform dulcitol broth enrichment as the first step in processing screening specimens

☐ Yes

☐ No

1. Which method for processing screening swabs do you use? Please check all that apply

☐ Chromogenic agar : specify

☐ Micronostyx Colorex Candida Plus / CHROMagar Candida Plus

☐ Micronostyx Colorex Candida / CHROMagar Candida

☐ BBL™ CHROMagar™ Candida

☐ Thermofisher Brilliance™ Candida

☐ Bio-Rad CandiSelect

☐ bioMerieux CHROMID Candida

☐ HARDYCHROM Candida

☐ Sabouraud agar

☐ Dulcitol salt agar

☐ PCR, commercial, specify product: Click here to enter text.

☐ PCR developed in-house, specify adapted protocol

☐ Other, specify

If ‘Chromogenic agar’, ‘Sabourad agar’ or Dulcitol salt agar are selected, Q14a and Q14b will appear:

14a. What temperature do you incubate plates at?

☐ 30°C

☐ 35-37°C

☐ 40-42°C

☐Other, please specify:

14b. How many hours do you incubate plates for before you call them negative?

[Open text]
